# Nature, smells, and human wellbeing

**DOI:** 10.1007/s13280-022-01760-w

**Published:** 2022-07-18

**Authors:** Phoebe R. Bentley, Jessica C. Fisher, Martin Dallimer, Robert D. Fish, Gail E. Austen, Katherine N. Irvine, Zoe G. Davies

**Affiliations:** 1 Durrell Institute of Conservation and Ecology (DICE), School of Anthropology and Conservation, University of Kent, Canterbury, CT2 8NR UK; 2 Sustainability Research Institute, School of Earth and Environment, University of Leeds, Leeds, LS9 2JT UK; 3 Social, Economic and Geographic Sciences Department, James Hutton Institute, Craigiebuckler, Aberdeen, AB15 8QH UK

**Keywords:** Environment, Green space, Memory, Olfactory, Sensory, Woodlands

## Abstract

The link between nature and human wellbeing is well established. However, few studies go beyond considering the visual and auditory underpinnings of this relationship, even though engaging with nature is a multisensory experience. While research linking smell to wellbeing exists, it focuses predominantly on smells as a source of nuisance/offence. Smells clearly have a prominent influence, but a significant knowledge gap remains in the nexus of nature, smell, and wellbeing. Here, we examine how smells experienced in woodlands contribute to wellbeing across four seasons. We show that smells are associated with multiple wellbeing domains, both positively and negatively. They are linked to memories, and specific ecological characteristics and processes over space/time. By making the link between the spatiotemporal variability in biodiversity and wellbeing explicit, we unearth a new line of enquiry. Overall, the multisensory experience must be considered by researchers, practitioners, policy-makers and planners looking to improve wellbeing through nature.

**Supplementary Information:**

The online version contains supplementary material available at 10.1007/s13280-022-01760-w.

## Introduction

Nature plays an integral role in promoting human health and wellbeing. This positive link has been documented across diverse environmental settings, including coasts (Wheeler et al. 2012; White et al. 2013; Britton et al. 2020), woodlands (O'Brien 2005; O'Brien and Morris 2014; Goodenough and Waite 2020) and urban parks (Irvine et al. 2013; Larson et al. 2016; Wang et al. 2016; Wood et al. 2017; Hunter et al. 2019). With the prevalence and associated costs of poor mental health and non-communicable diseases rising globally (Foreman et al. 2018; WHO 2020), the positive impacts of nature have become of significant interest to healthcare (e.g. World Health Organisation; WHO 2016) and environmental policy (e.g. UK Government Department for Food and Rural Affairs, DEFRA; Garside et al. 2020), and practice sectors (e.g. The Wildlife Trusts; Rogerson et al. 2017). Yet, there remains a paucity of evidence investigating precisely which attributes of nature could affect human wellbeing and why.

Nature is a multisensory experience (Franco et al. 2017). Indeed, natural environments can provide a diversity of stimuli via different ecological characteristics and processes, as well as seasonal changes (Pálsdóttir et al. 2021), thus enhancing the multisensory experience. To date, the colours, shapes and other visual aspects of nature have been the primary research focus. However, interest in sound is also growing (e.g. Irvine et al. 2009; Hedblom et al. 2014; Bates et al. 2020). The stimulation of multiple senses may simultaneously result in additive or cumulative benefits (Dijk and Weffers 2011; Hedblom et al. 2019; Spence 2020). For instance, Aristizabal et al. (2021) showed that in a workplace environment, the combined effects of visual and auditory enhancement (nature visuals and sounds, respectively) for improved wellbeing were more powerful than each component individually. In a virtual experiment that relied on visual stimuli, participants reported that they missed the full sensory experience of being in nature (Kjellgren and Buhrkall 2010). More recent studies also show that while simulated experiences of nature can reduce stress, stress reduction is greater *in situ* (White et al. 2018; Lahart et al. 2019). Assessing the impact that all individual senses have on nature-wellbeing outcomes, and how they fluctuate across space and time, is therefore needed to optimise the design and management of green/blue spaces, as well as improve the targeting of nature-based health interventions (Franco et al. 2017).

While the framing of many environmental issues has increasingly moved away from a traditional emphasis on the adverse effects on society towards the potential benefits to people, this has not been the case for smells. Research linking smell to health/wellbeing predominantly considers anthropogenic smells as a source of nuisance or offence, and has focused on identifying interventions to mitigate them. For example, in an early review of smells, Engen (1986) highlights odours as a source of irritation, inducing mental fatigue and even pathological outcomes. The impacts of odours from sewage or landfill sites on the wellbeing of workers and nearby communities has been rigorously assessed (Dzaman et al. 2009; Heaney et al. 2011; Sakawi et al. 2011; Fujiwara et al. 2020). Landscapes of smell (‘smellscapes’) of emissions, food, tobacco and waste have been developed for major cities, including Barcelona and London, to aid urban planning (Quercia et al. 2015).

Nonetheless, the use of smells to promote health and wellbeing has occurred throughout history, being central to traditional and contemporary aromatherapy techniques (Schloss et al. 2015; Hickman 2021), as well as contributing to the therapeutic benefits of forest bathing (short, leisurely visits to a forest for relaxation; Li 2010). Therapeutic horticulturalists report the psychological and physiological benefits of plant smells such as plum blossom (*Prunus mume*) (Jo et al. 2013) and geraniums (Order: Geraniales) (Pálsdóttir et al. 2021). Such evidence can be used to inform the design of salutogenic (health-promoting) sensory gardens, particularly beneficial for those with visual or hearing impairments (Bell 2020). The role of natural environments for good health and wellbeing was particularly apparent during the COVID-19 pandemic. Many people, particularly those from deprived backgrounds, became reliant on public green space when national lockdowns were imposed (Natural England 2020). As a result of the viral infection, lack of smell (known as ‘anosmia’) became prominent, and was shown to impair people’s quality of life and emotional wellbeing (Burges Watson et al. 2021). The use of smells to evoke memories has been employed for the treatment of a series of neurological disorders, including Alzheimer’s disease (Glachet et al. 2019), and has been shown to increase positive emotions, enhance feelings of comfort and happiness, and decrease anxiety (Matsunaga et al. 2011). A significant body of evidence demonstrates links between the olfactory system with metabolic health (Riera et al. 2017), mood (Goel and Grasso 2004), cognition (Lorig et al. 1990), memories (Horowitz 2011), behaviour (Millot and Brand 2001) and autoimmune conditions (Strous and Shoenfeld 2006). Indeed, the prominence of smells for our wellbeing has only recently been acknowledged by scientists (McGann 2017; Spence 2021a).

Researchers across fields of sociology, economics, anthropology, medical history and human geography have highlighted the sociocultural context and construction of smells, including the role they play in building individual identities, sociality and how we understand and interact with the world around us (e.g. Synnott 1991; Low 2013; Cerulo 2018; Hickman 2021). For instance, the perfume industry heavily markets floral scents, including rose (Mileva et al. 2021) and lavender (Aburjai and Natsheh 2003). Smells have been shown to influence people’s perceptions of, and the values they assign to, different places, objects and people (Li et al. 2007; Camps et al. 2014). In particular, economic sectors such as retail and tourism use smells to manipulate consumer behaviour (Spence et al. 2014; Ali and Ahmed 2019; De Luca and Botelho 2019).

Research into understanding the sensory influence of the natural environment is hindered by methodological challenges. Vision is considered to be well understood and measurable in biological science, with a plethora of established ‘off-the-shelf’ technologies and approaches to study it (Hutmacher 2019). Likewise, sound can be measured by vibrations, and touch can be described by pressure (Synnott 1991). In contrast, there is little consensus over how to classify and measure different smells. People also find it to harder to talk about smells compared to other senses, possibly due to the complexity of neurological pathways between the input of smells and the output of verbalised language (Olofsson and Gottfried 2015). These factors make smells challenging to investigate and, consequently, it is arguably the least understood sense.

## Theoretical Framework

In evolutionary terms, the olfactory system plays a critical role in identifying food, mates, and predators, as well as detecting warning signs for dangers like spoiled foods or chemical hazards (Sowndhararajan and Kim 2016). Smell is unique amongst the senses in terms of its biological mechanism. Generally, the senses (e.g. sight, sound) first travel to a brain region called the thalamus, which acts as a switchboard to relay information about the things we see, hear or touch, to the rest of the brain (Torrico and Munacomi 2020). However, smells travel directly to the limbic system that houses the amygdala, where we process emotions, and the hippocampus, where our learning and memory formations occur (Firestein 2001). This strong connection between smells and our emotions can be observed by the manifestation of depressive episodes following the development of olfactory disorders (Soudry et al. 2011). Similarly, olfactory-based memories can induce a powerful emotional response when triggered (Tischler and Clapp 2020).

Existing evidence suggests that smells associated with nature can improve human wellbeing. While investigating the therapeutic potential of different landscapes, Finlay et al. (2015) found that participants enjoyed the smell of herbs and had a desire to immerse themselves in smells. In an *ex situ*, lab-based study, Glass et al. (2014) discovered that summer air (leaf alcohol) and beeswax were associated with promoting happiness. Additionally, Hedblom et al. (2019) demonstrated that smells may be better at reducing stress than visual stimuli via a virtual experiment set within an urban context. This work is compelling, but partial. For instance, we do not know how smells influenced wellbeing in the work of Finlay et al. (2015). In Glass et al. (2014), only a small number of ‘pleasant’ smells (summer air, candles) were compared to extreme ‘unpleasant’ smells (disinfectant, vomit), and the authors used synthetic chemical mixtures to represent the smells. Hedblom et al. (2019) focused purely on stress reduction. Moreover, these studies collectively measure how smells act upon different individual domains of human wellbeing, which is itself a multidimensional construct (Engel 1977; Andrews and McKennell 1980; Fava and Sonino 2008; Linton et al. 2016).

Originally developed in medicine (Engel 1977), researchers have investigated the ‘biopsychosocial’ model of wellbeing, representing the physical (‘bio’), psychological (‘psycho’) and social domains (Engel 1977; Fava and Sonino 2008). This model has been adopted by a recent theoretical framework examining the relationship between biodiversity and human wellbeing (Marselle et al. 2021), which proposes that particular traits (e.g. smells) can influence these wellbeing domains via several pathways (e.g. restoring capacity), while taking account of various moderators (e.g. weather). However, psychological wellbeing is also thought to encompass separate cognitive and emotional domains (Andrews and McKennell 1980), while an expanded version of the biopsychosocial model recognises a spiritual wellbeing domain (McKee and Chappel 1992; Linton et al. 2016; Irvine et al. 2019). As such, the ‘biopsychosocial-spiritual’ model of wellbeing may comprise physical (outcomes related to the physical body and how one feels physically), cognitive (influences on a person’s state of mind), emotional (refers to the presence of positive and negative emotions and mood), social (how an individual perceives their connections with others) and spiritual (involves the relationship with one’s self and in some cases feelings of being connected to something greater) domains (Irvine et al. 2013; Linton et al. 2016). Applying this model to nature-health research is needed to help transcend disciplinary boundaries, as well as align findings between different studies through a common language and theory (Irvine et al., 2013).

Smells clearly have a prominent influence on human health, but a significant knowledge gap remains in the nexus of nature, smell, and human wellbeing. Here, we undertake an exploratory study, taking participants to two British woodlands across four seasons, to examine how the smells people experience *in situ* are linked with the various wellbeing domains. Our findings move beyond the control of nuisance odours and provide a basis for more targeted future research in a number of disciplines. Moreover, our findings offer important insights into how policy-makers and practitioners could best manage environmental spaces to promote human wellbeing.

## Materials and Methods

### Study sites

This study focused on woodlands, which represent 13% of UK landcover (Forest Research 2020) and occur country-wide both inside and outside of urban areas. They are the most visited green space behind ‘urban parks’ and ‘paths, cycleways & bridleways’ in England (Natural England 2019), and are considered the second most wellbeing-enhancing type of natural environment after beaches (Harrison et al. 2014). We selected two woodlands located in central England. The first, Sherwood Forest (Figure S1a-d; 53.2059° N, 1.0861° W), is an ancient woodland dominated by oak (*Quercus robur*). The second, Clumber Park (Figure S1e–h; 53.2679° N, 1.0639° W), is a managed mixed deciduous and coniferous plantation woodland. Using these different habitats allowed participants to encounter a variety of ecological stimuli.

### Participants

A total of 194 individuals were recruited via a social research company to attend one of four workshops (one workshop per season; Table S1). Participants were purposively selected to maximise the diversity of sociodemographic backgrounds and perspectives. This was done according to gender (a balance across those identifying as male and female), age (balance across three brackets of 18–29, 30–59 and > 60 years old), a mix of ethnicities (> 20% non-white British), a mix of people from the different regions (across England, Wales and Scotland), individuals from varying social grades, and a mix of both urban and rural dwellers (> 20% of individuals from rural locations).

### Data collection

The four workshops were conducted in February, April, June and October 2019. During each workshop, participants visited Sherwood Forest in the morning and Clumber Park in the afternoon. Upon arrival, participants were told that they would be taking part in a ‘*woodland scavenger hunt*’. Participants were invited to ‘*look around and notice different elements of the woodland*’ and write down what they noticed in terms of colours, textures, sounds, shapes, and smells, and were reassured that they did not need to identify particular species. We asked participants to focus on the natural attributes of the woodland (e.g. biodiversity), rather than dogs, other people or artificial objects. Participants were also asked to indicate whether they liked, disliked or felt indifferent towards each attribute. They were given an hour to complete the task at each location and were asked to undertake it individually.

Following the visit to each woodland, participants were divided into groups, each comprising 10 individuals. These focus groups discussed the woodland attributes identified by participants, beginning with the question ‘*what was your general impression of this woodland?’*, followed by a loosely structured dialogue around whether they liked, disliked or were indifferent to the attribute and why (Appendix S1). Care was taken throughout all activities not to indicate a specific interest in wellbeing. Each participant provided their informed consent and ethical approval was gained from the University of Kent’s School of Anthropology and Conservation Research Ethics Committee (Ref: 009-ST-19).

### Data analysis

All discussions were audio recorded and transcribed verbatim, with the transcripts imported into NVivo (Version 12, QSR International Ply Ltd.). We then conducted a qualitative content analysis, working inductively to create thematic codes that described the ways in which smells were discussed and interpreted by participants. First, all dialogue relevant to smells was identified, as well as reasoning that stated or implied relationships between smells and wellbeing. During this process it became apparent that the discussions fell into one of two categories: (i) specific named smells, where a particular scent had been identified; and (ii) a perceived absence of smell, where the individual had referred to a relative absence of smell or fresh air. We also identified instances when smells had triggered memories. Second, we used the biopsychosocial-spiritual model of wellbeing to provide theoretical grounding for our findings (Irvine et al., 2013). This integration with theory allowed us to move beyond descriptive codes, and identify physical, cognitive, emotional, social, and spiritual domains of human wellbeing, based on the way people described their experiences. References to ‘global’ wellbeing (unspecified sense of overall health/wellbeing or lack thereof) also emerged in the data, which was subsequently included as an additional code following Irvine et al. (2013).

Where transcript text was coded as smell, we extracted mentions of specific named smells, mirroring the language used by participants (e.g. “*woody*”, “*earthy*”, “*floral*”). This abstraction of the data allowed us to capture patterns in which specific smells were noticed, and how frequently. Specific named smells were aggregated if the terms were synonymous (e.g. combining “*decaying”* with “*decomposition*”) or had alternate endings (e.g. combining “*earthy”* with “*earthiness*”). Likewise, “*couldn’t smell anything*” and “*devoid of smells*” were grouped into absence of smell. We then quantified patterns in the frequency of specific named smells across the four seasons for both woodlands separately. We also quantified how the contribution of smells to each domain of human wellbeing varied across the seasons.

## Results

Across all four seasons, participants made 337 mentions of smells in the two woodlands (compared to 223 for sound, 494 for textures, 596 for colours and 786 for shapes). Participants’ references to smells used descriptors that pertained to both specific named smells (e.g. “*earthy*” and “*mouldy*”), but also highlighted a perceived absence of smell. There was a considerable narrative around “*fresh air*” which, when prompted to elaborate, was characterised by a distinct absence of smell. This was frequently conveyed as a contrast to urban areas. For instance: “*there is no smell so it’s clean, you know. If you’re in the city, for instance, you can smell the traffic*”. Another participant described this as: “*I’m currently living in a big city and you go out there and you can smell all the different things, food, near the shops and all that, and actually having like sort of a nothing but fresh smell, especially when it’s cold, I think it’s more noticeable*”. There were 103 unique specific named smells mentioned by participants (Table S2).

### Multiple wellbeing domains can be impacted by smells

**Fig. 1:**
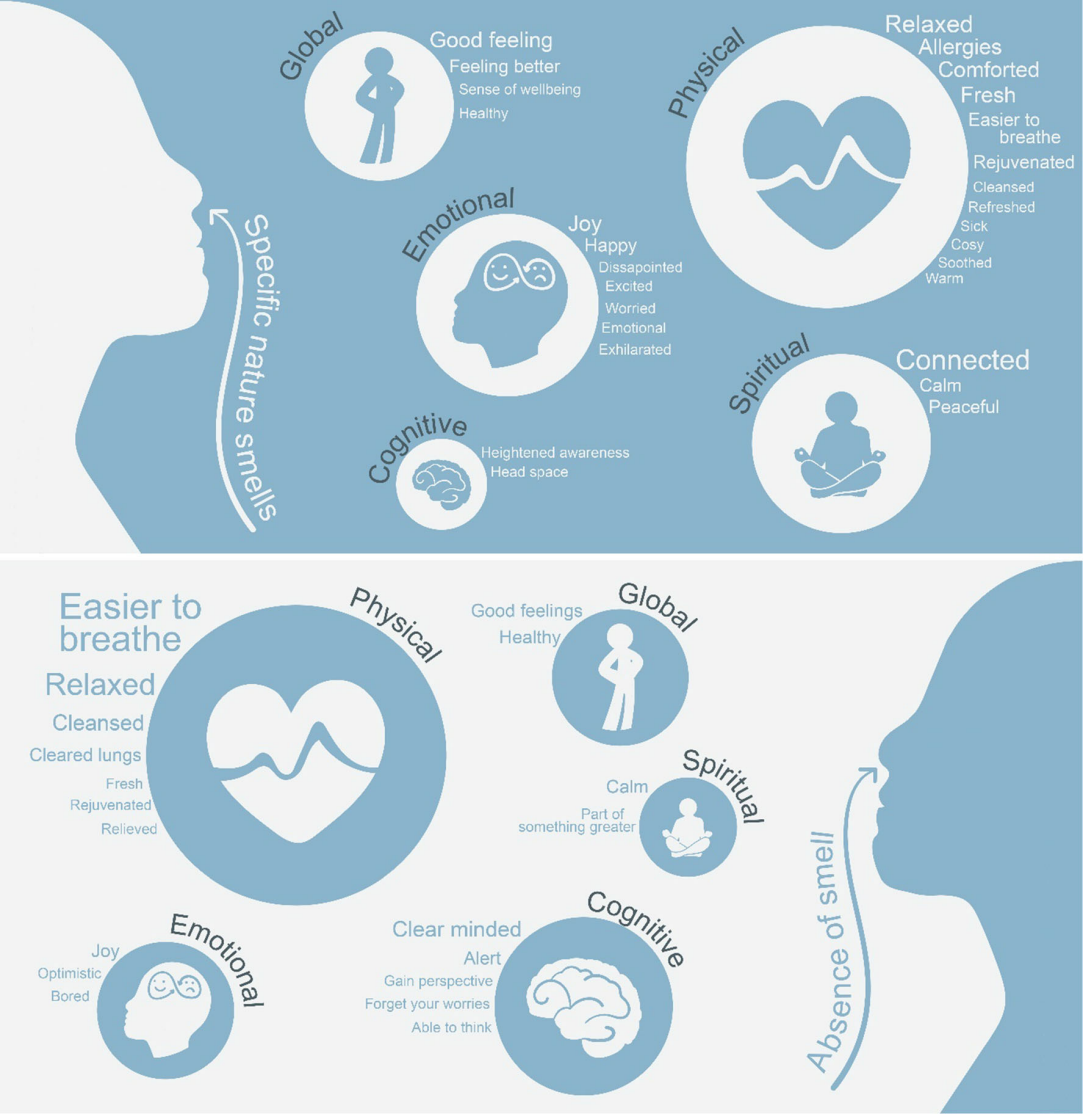
Graphical depiction of the wellbeing effects experienced by participants (*N* = 194) following an encounter with specific smells or the perceived absence of smell during visits to Sherwood Forest and Clumber Park from winter to autumn 2019. The wellbeing domains examined comprise physical, emotional, cognitive, spiritual, social or global wellbeing, although no smells were found to relate to social wellbeing. The size of the circle denotes the proportion of participant comments that relate to each wellbeing domain. The sub-codes reflect the language used by participants to describe the wellbeing effect, and the size of the sub-code text is proportional to the amount of times it was mentioned

Both specific named smells and a perceived absence of smell were identified as influencing multiple domains of wellbeing (Fig. 1). Of the 337 smell-related comments, 31% described experiencing smells with an impact on physical, cognitive, emotional, spiritual, or global wellbeing. Social wellbeing was not discussed at all, for either woodland or in any season.

#### Physical wellbeing

Physical wellbeing was the most frequently cited domain with regard to both specific smells that individuals experienced and perceived lack of smell. The effects were reported as both adverse and favourable. Participants most frequently noted how specific smells evoked feelings of relaxation. One individual attributed this to “*earthy, woody smells*”, describing how they “*wanted to just switch off for relaxation*”. Such smells were also associated with feelings of comfort and rejuvenation. Likewise, narratives around relaxation were prominent in relation to an absence of smell, for example: “[I] *just relaxed. Really relaxed. You’re just breathing it* [fresh air] *in and it’s lovely. Clean out all the smog from the towns where you’ve been living.*”

For some, the smells were deleterious to wellbeing: “*Gypsy rose. I could smell that. It kind of makes my nose itch and eyes itch*”. Other participants explicitly stated that smells warn them about the presence of allergen-causing substances. This was summarised by one participant: “*Because when you can actually smell pollen…even if it’s not the pollen that sets me off, I smell it and kind of think, ‘Oh, it’s going to be that time of year soon’*”.

#### Cognitive wellbeing

Cognitive wellbeing was linked to specific smells by a small number of participants. For one individual a “*heightened awareness*” was induced by “*pleasant, floral*” smells. Another described how: “*if you need some head space just go and just sit and absorb the smells*”. Interestingly, cognitive wellbeing impacts were more frequently discussed in response to an absence of smell. For example: “*the cleanliness and the freshness of the air around you clears your mind*”. It was further noted that the lack of overpowering smells, such as one would encounter in an urban environment, allowed the senses to be cleared and individuals were therefore able to take in other stimuli in the environment. This sentiment was captured well by the following: “*I think because nothing is really overpowering your senses, you can focus on something visually.* […] *while your senses are being* [overpowered], *that’s always on your mind and you can’t really appreciate other smaller things. So that clearness allows you actually look and search for some visual stuff. If I found a place very offensive to smell I might not be able to get over that point and really appreciate the surroundings and listen to the sounds or the visuals*.”

#### Emotional wellbeing

A range of positive emotional states, including joy, happiness and excitement, were discussed in relation to specific smells. In the case of pine, this response was attributed to an association with Christmas: “*I smelt pine, and that cheered me up because, for some reason, it makes me feel happy* […] *It’s Christmas*”. Nonetheless, certain smells evoked undesirable emotions. For instance, one individual suggested that the “*overriding smell*” of ferns made them fearful that there may be ticks (small, parasitic arachnids that can bite human beings) present.

#### Spiritual wellbeing

Participants commonly described a sense of connecting to nature and the wider world around them in response to a specific smell. “*Earthy*” smells featured heavily in this relationship, illustrated by comments such as: “*to me you connect with nature and that smell* [earthy] *is really sort of powerful. To me it’s not damp. It is quite powerful”* and: “*you’ve still got that earthy smell and you know that underneath that they’re not dead. You’ve got all the roots of the trees, as you said, connecting you*”. Feelings of calmness and peace were also conveyed, again linked to damp or earthy smells: “*It’s a peaceful smell to me, yeah. It makes me feel peaceful*”.

#### Global wellbeing

Comments that represented an overall sense of wellbeing were evident across both specific named smells and an absence of smell. Once again, “*fresh dampness*” and “*earthy*” smells were predominant. One participant described their global wellbeing: “*you get a good feeling, like it’s natural earth and water and the wind, the three together, earth, water and air*”. Although less frequent, global wellbeing sentiments were highlighted in response to the perceived absence of smell. This was generally linked to good feelings or feelings of being healthy. As with other wellbeing domains, examples were often communicated by comparing their experience in woodlands to an urban environment: “[I] *just like how fresh and clean it felt. I guess because the air was slightly cold it just felt like so healthy to breathe it in. I guess like living in a city as well it’s like you don’t really get that, and I don’t get to get outside in the countryside that much, but how nice it felt to like not really smelling anything.*”

### Smells experienced in nature evoke memories

We revealed a strong link between specific smells experienced in nature and memories. Most of the events described by participants in this study were not however tied to the woodlands visited, with few referring to woodland at all. Instead, smells evoked memories related to childhood activities: “*I got kind of a classic leaf mould smell. It was one of those smell-to-memory moments where, you know, I was suddenly back in the back garden with my Dad and I’m eight and he’s turning over the compost heap on an autumn morning on a Sunday. You know, I’m helping him out. So it was kind of quite an emotional moment, sort of getting that kind of connection.*” Individuals appeared to create meaningful connections with particular smells, rather than specific places, and associate this with a memorable event. This, in turn, appeared to influence wellbeing by provoking emotional reactions to the memory.

Memories developed in adulthood were less commonly mentioned. Instances of these tended to focus on activities occurring beyond woodlands, with descriptions often less tied to a personalised event and more generalised: “*It properly smelt* [woody] *– when you got your dry wood set up for a log fire and there’s something dead comforting about that, and then you can hear it crackling underneath. It sounds a bit like fire crackling. So it was a nice little nostalgic moment.*” and: “*It evokes lots of lovely feelings, because it’s Christmassy and it’s that smell of pine. I love that smell of pine. Well for me, I think it’s a lovely fresh smell. I always associate the smell of pine and pinecones with quite a fresh sort of menthol smell which I really like. Yeah, and it just, yeah, it evokes memories of Christmas time and that sort of time of year for me.*” The triggering of memories in adulthood therefore induced emotions, providing a connection to past events.

### Profiles of smells across space and time

Using the specific named smells, we compared how the olfactory profile of each woodland was discussed (Table 1). While both woodlands were considered as “*damp*” and “*fresh*”, Sherwood Forest was described with smells like “*musty*”, “*decay*” and “*foliage*”, while participants smelled “*mud*” and “*pine*” in Clumber Park. After summing the frequency of specific named smells noted by participants, we found Sherwood was described using 34% more smells than Clumber Park.

**Table 1:** The frequency of smells mentioned by workshop participants (*N* = 194) during discussions about their *in situ* experiences of Sherwood Forest and Clumber Park from winter to autumn 2019. Only those smells that were mentioned four or more times across workshops are included in the table, listed in order of most to least frequent

Woodland	Smells
Sherwood Forest	*damp, absence of smell, fresh, fresh air, clean, earthy, musty, nature, smell, wood, decay, crisp, cold, foliage, smells, wet*
Clumber Park	*absence of smell, damp, fresh, earthy, smell, fresh air, clean, rain, mud, nature, pine, smells, weak smell*

Alongside smells evoking particular seasonal memories (e.g. the smell of pine induced memories of Christmas), smells in the woodland also stimulated thoughts and ideas about seasonality, such as “*earthy*” smells and the spring emergence of new shoots: “*Dying off now I suppose but you’ve still got that earthy smell and you know that underneath that they’re* [the trees] *not dead. You’ve got all the roots of the trees, as you said, connecting and you can see the bark of the tree. So alive* […] *and thinking it’s almost hibernating for the winter but you know it’s there and it’s going to come back up in the spring.*” Woodland smells also elicited a sense of the changing seasons, and participants often described each season as having its own typical smell: “*It’s just the changing of the seasons I feel. You know, it’s nice to have the difference isn’t it? The summer smells, autumn smells. Not so much the winter smells. I don’t like the cold either. But yes, it’s nice to have the different seasons and the smell of each season. It’s just nature again and things change. Just taking it all in and seeing how nature reacts and what happens.*”

**Fig. 2:**
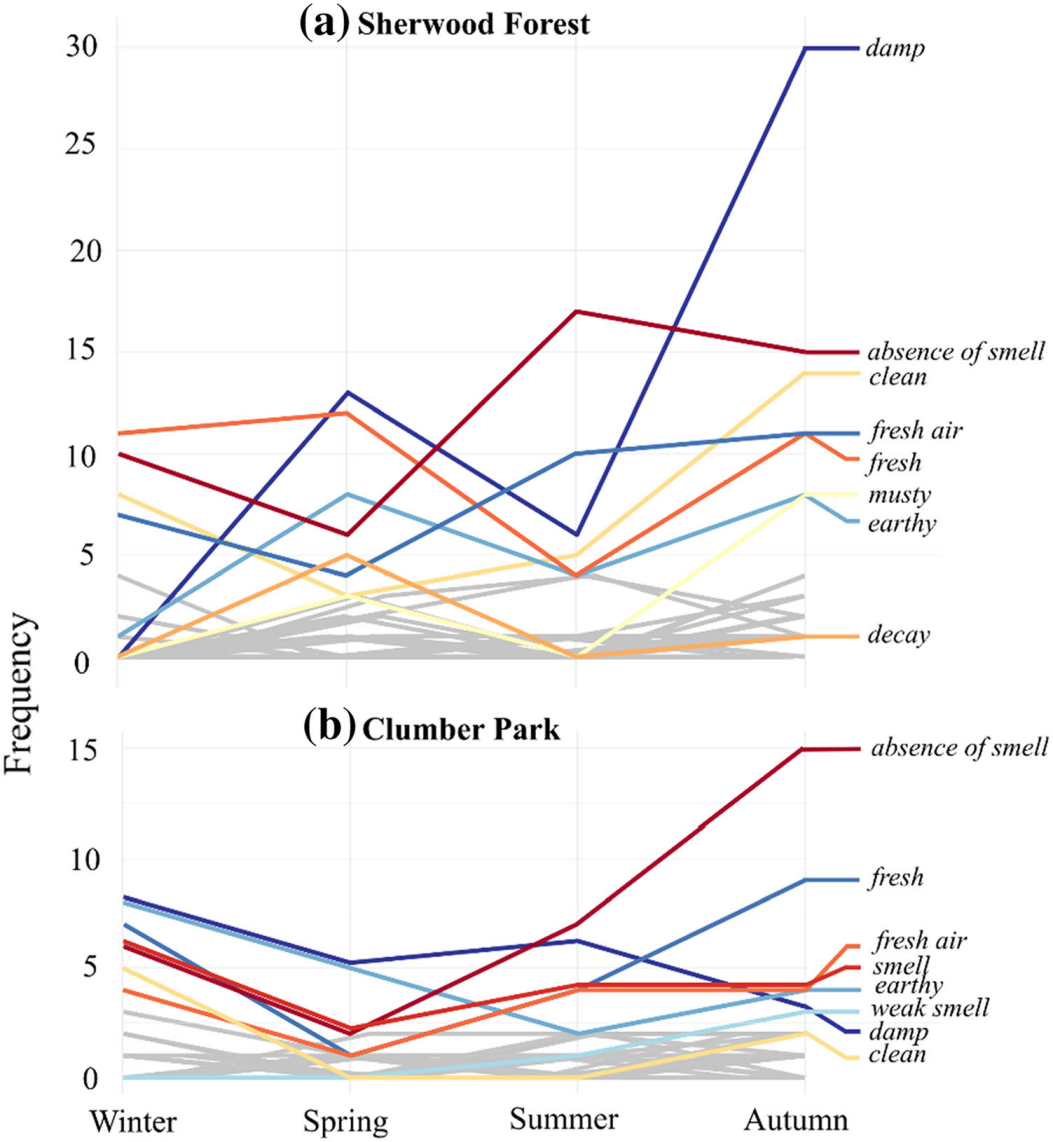
The frequency of named smells reported by participants (*N* = 194) visiting Sherwood Forest and Clumber Park from winter to autumn 2019. The most frequently discussed smells are indicated by coloured lines, with other, less frequently reported smells visible in grey

We used the specific named smells to quantitatively examine how smells changed across the seasons for each woodland (Fig. 2). During winter, “*earthy*” and “*nature*” smells were replaced by descriptions of an “*absence of smell*” and “*fresh*” in Sherwood Forest, while “*earthy*” and “*damp*” smells were more frequently referred to for Clumber Park. With the arrival of spring, participants continued to report “*fresh*” and “*absence of smell*” in Sherwood Forest, but also more “*damp*” and “*earthy*” smells, which were also mentioned most in Clumber Park. In summer, there was an increase in descriptions of “*fresh air*” and “*absence of smell*”, with equal prevalence of “*damp*” smells, for both woodlands. Moving into autumn, participants predominantly described Sherwood Forest as “*damp*”, although mention of “*absence of smell*” and “*clean*” were also prevalent, whereas Clumber Park was most often described with “*absence of smell*” and “*fresh*”.

**Fig. 3:**
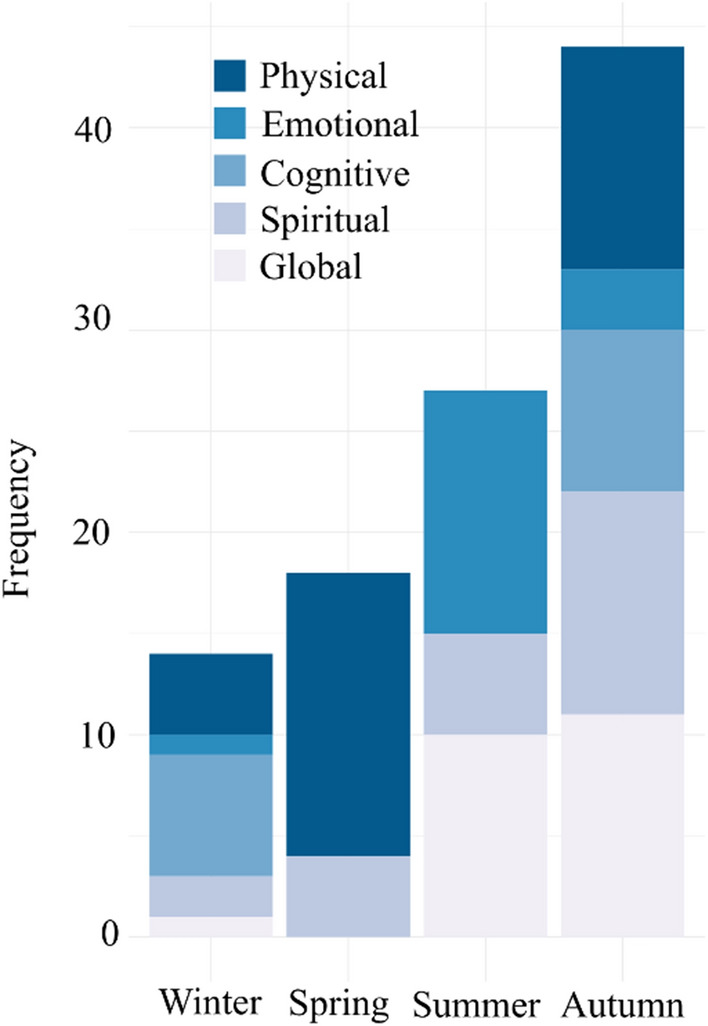
The contribution of smells to different domains of wellbeing (physical, emotional, cognitive, spiritual and global) reported by participants (*N* = 194) visiting Sherwood Forest and Clumber Park from winter to autumn 2019

When assessing the contribution of smells to each wellbeing domain across the seasons, they were markedly different (Fig. 3). For example, specific named smells were used the least in participants’ descriptions of winter, although they related to all wellbeing domains with the exception of social wellbeing. In spring, smells made a particular contribution to the physical and spiritual domains of wellbeing, while in summer smells were most often linked to emotional, spiritual and global wellbeing domains. In autumn, participants used the most smells when describing their woodland experience, with contributions mentioned to each wellbeing domain with the exception of social wellbeing.

## Discussion

Unlike other senses, smells are unique in the mechanism with which they affect cognitive processes, and subsequently our emotions, memories, and perceptions of the world around us. In a woodland setting, smells and perceived absence of smell are a notable part of the nature experience, contributing to a variety of human wellbeing domains. These findings highlight the need for researchers working in the nature-health nexus to consider that experiences are likely to be multisensory, and that different domains of wellbeing can be influenced by smells. Such specificity will enable better-informed recommendations for landscape planners and public health specialists to optimise the benefits provided by the natural environment for specific wellbeing outcomes.

We found smells affected multiple domains of wellbeing with physical wellbeing discussed most frequently, particularly in relation to relaxation, comfort, and rejuvenation. Even absence of smell was perceived to improve physical wellbeing, providing a cleansing service, removing the pollution and unwanted smells associated with urban areas, therefore enabling relaxation. Relaxation reduces stress and lowers cortisol levels (Unger et al. 2017). High stress levels are a risk factor in a multitude of diseases, representing a significant global public health concern (VanItallie 2002; Nielsen et al. 2005; Bisht et al. 2018). Experiencing smells in nature alone is unlikely to mitigate such risks, but small interventions on a large scale could, nonetheless, deliver significant public health benefits (Indig et al. 2018). Woodland smells also stimulated positive emotional wellbeing, which is linked to measurable physical health outcomes (Pressman et al. 2019). For instance, greater levels of positive emotion are linked to longer lifespans (Zhang and Han 2016), better prognosis for diseases, including cancer and HIV (Hernandez et al. 2018), as well as cardiovascular fitness (Boehm and Kubzansky 2012). Indeed, participants reported higher global wellbeing, in terms of ‘feeling better’ and ‘healthy’, after encountering woodland smells, as well as the absence of smell linked to urban environments. Further enquiry might interrogate the role of non-natural smells that we asked participants explicitly not to focus on (e.g. dogs, people, artificial objects) as these are particularly common in urban environments, and conducting the same research protocol elsewhere would elucidate whether the same smells (e.g. flowers) in different settings (including urban environments) elicit similar wellbeing responses. Nevertheless, it is clear that smells experienced in nature evoke positive physical, emotional, and global wellbeing, which could result in quantifiable improvements to people’s health and wellbeing.

Smells also related to the cognitive and spiritual domains of human wellbeing. Participant descriptions of smells in relation to cognitive wellbeing represented the concept of a restorative natural environment, specifically the notion of ‘soft fascination’ (Kaplan 1995). Softly fascinating stimuli allow people to rest their directed attention, providing an opportunity to restore cognitive capacity (Kaplan 1995), as research on visual (e.g. Van den Berg et al. 2016) and auditory nature attributes (e.g. Cerwén et al. 2016) has shown. Specific woodland smells (e.g. ‘earthy’) also evoked feelings of peace and calmness, and participants often referred to a connection with nature or a transcendent other, falling within the theorised framework for how biodiversity relates to spiritual wellbeing (Irvine et al. 2019). These findings align with those of Pálsdóttir et al. (2021), who document how garden smells (e.g. citrus) evoke feelings of peace and calmness, although the authors do not explicitly classify this as spiritual wellbeing. Considering the varied ways by which smells can impact different wellbeing domains could help improve study designs that seek to measure specific outcomes from interventions.

We also found a clear link between woodland smells and participants’ memories. Scents are especially effective at inducing autobiographical memories, lasting longer than any other sensory memory (Miles and Jenkins 2000) and providing a better cue to recall than other senses (Chu and Downes 2000; Herz 2016). A link to memories could signal a contribution of smells to place attachment (Milligan 1998; Lewicka 2011), which is an emotional link to two interwoven components: (i) interactional past, concerning the memories of interactions with a place; and (ii) interactional potential, addressing future experiences that are likely or possible to occur at a place. Memorable events become associated with specific sites and these experiences contribute a sense of meaning, often generating compelling reactions. Smells that induce memories (‘reminiscence therapy’) can support the retention of identity in dementia sufferers (Rathbone et al. 2019; Tischler and Clapp 2020), improve cognitive function, and reduce depressive symptoms (Cui et al. 2017). Non-pharmacological smell therapy interventions that incorporate woodland visits could therefore benefit individuals with a wide range of mental health conditions. Sensory therapy in natural surroundings is more effective than equivalent therapy within standard treatment rooms (Goto et al. 2014), and could be used by education professionals to enhance the diversity of experiences of schoolchildren, establishing a basis upon which individuals may reflect on their memories in later life (Beery and Lekies 2021).

Analysing participants’ language allowed us to capture detailed information about specific ecological characteristics and processes. While it is possible that the time of day influenced what smells were noticed (with each woodland visited at the same time each season) (cf. Spence 2021b), the smells named by participants were characteristic of each woodland type. Smells like ‘musty’ and ‘decay’ in Sherwood Forest reflect its ancient woodland status, typified (in the UK) by abundant fungi and decomposers (Glaves et al. 2009). This was further reflected in seasonal patterns, particularly as the weather was typical for each season. For instance, the weather during the winter workshop was cold and frosty, with clear skies. Sherwood Forest was described mainly as ‘fresh’ and with an ‘absence of smell’ (as a result of the frozen ground), while in autumn it was described as ‘damp’, when warm and wet weather accelerates decay and decomposition (Boddy and Swift 1984). This was different to Clumber Park in autumn, where the woodland was described as comparatively less damp and predominantly fresh. It is likely that the prominence of coniferous trees in Clumber Park may have led to lower levels of leaf senescence and productivity on the woodland floor (Richardson et al. 2010), thus producing less smells. Across the two woodlands, participants discussed relatively fewer smells in winter compared with subsequent seasons. Aside from reduced ecological activity, people’s olfactory senses may have been temporarily subdued due to seasonal viruses (Pellegrino et al. 2017). Indeed, smells have been shown to be less prominent in people’s descriptions of gardens in winter (Pálsdóttir et al. 2021). Nonetheless, during winter participants made links to culturally and seasonally specific memories (e.g. pine scent with Christmas), thus facilitating the contribution of winter smells to multiple wellbeing domains. These findings demonstrate how specific ecological characteristics and processes elicit wellbeing responses. By making the link between the spatiotemporal variability in biodiversity and psychological wellbeing explicit, we unearth a new line of enquiry in nature-wellbeing research.

Social wellbeing was not an outcome of the participants’ experience of woodland smells. Other studies on nature and wellbeing that have situated groups of participants in woodlands have documented improved social connections and support, often through the absence of distractions that might be usual in an urban setting (Warber et al. 2015). Given that participants in our study were completing tasks individually, there was less opportunity for social interaction, and thus the absence of social wellbeing may be an artefact of the study design. Nonetheless, discussions about the activities took place in focus groups, where smells could have acted as a stimulus for participants to relate their experiences to one another, or reflect on past social interactions and bonds facilitated by nature. Future research is needed to ascertain how participants derive social wellbeing from natural environments, comparing both individual and group-based activities.

## Conclusion

Smells play an important role in delivering wellbeing benefits from interacting with nature, and they are unique amongst the senses in how they are interpreted by the human brain. Smells influence multiple human wellbeing domains, often via a strong link to memory and specific ecological characteristics and processes that vary across space and time. Extrapolating beyond the language used by our participants and the woodland settings they experienced, this work opens opportunities for new lines of enquiry with important applied implications. Foremost, they indicate that not all experiences of nature are equal in terms of their contribution to human wellbeing, as they are closely interlinked with past experiences, the ecological setting and season. This is critical to informing and tailoring nature-based interventions to promote specific health and wellbeing outcomes, particularly at a time when social (and ‘green’) prescribing is rapidly gaining traction (Garside et al. 2020), and evidence supporting appropriate programme content development remains scarce (Shanahan et al. 2019). We unearth a wealth of opportunities to replicate our research protocol in different landscapes (e.g. city versus woodland), populations (e.g. rural versus urban) and life stages (e.g. children versus adults), to rapidly advance our understanding. For instance, it is likely that the smells that trigger specific wellbeing outcomes will differ across cultures and countries, given the importance of the sociocultural context of smell (e.g. Synnott 1991; Low 2013; Cerulo 2018; Hickman 2021). Such differences can be exposed by examining participants’ language and, in turn, the cultural and historical phenomenon that inform their personal experiences (Hickman 2021). To this end, we invite environmental psychologists, education professionals, therapeutic horticulturalists, landscape planners, conservation scientists and decision-makers to better recognise the wide-ranging (and potentially low-cost) benefits that the multisensory natural environment can bring to our health and wellbeing.

## Supplementary Information

Below is the link to the electronic supplementary material.

Supplementary file1 (PDF 891 KB)
