# Supplementary material for: Nature, smells, and human wellbeing
Source: Ambio. 2022 Jul 18;52(1):1–14. doi: 10.1007/s13280-022-01760-w (PMC9289359; doi:10.1007/s13280-022-01760-w)
Supplement: Supplementary file 1 — Supplementary file1 (PDF 891 KB) [file 13280_2022_1760_MOESM1_ESM.pdf]

Ambio

Supplementary Information

This supplementary information has not been peer reviewed

Title: **Nature, smells, and human wellbeing**

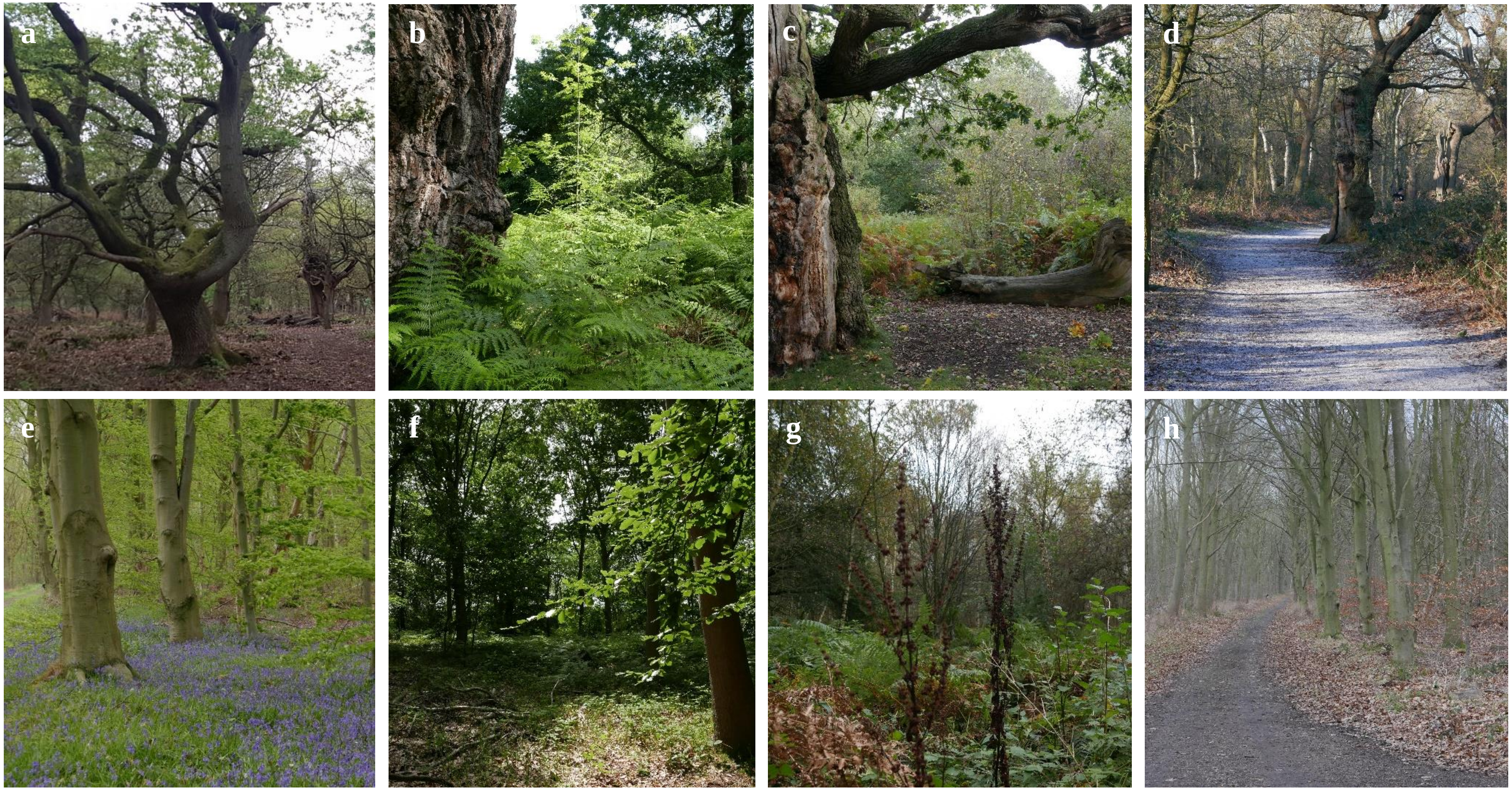
a
b
c
d
e
f
g
h

Figure S1. Workshop study sites. Participants visited two different woodlands in central England. Sherwood Forest is an ancient woodland characterized by oak trees depicted in (a) spring (b) summer (c) autumn and (d) winter. Clumber Park is a managed mixed deciduous and coniferous plantation woodland shown in (e) spring (f) summer (g) autumn and (h) winter..

Table S1. Sociodemographic/economic characteristics of the study participants. Individuals (*N*=194) were recruited via a social research company, and were selected purposively to maximise the diversity of backgrounds and perspectives across the British population.

Characteristic	n	%	Characteristic	n	%
Gender:			Employment status:		
Female	103	53.1	Employed	107	55.1
Male	90	46.4	Self-employed	24	12.4
Prefer not to say	1	0.5	Unemployed	9	4.6
Ethnicity:			Retired	36	18.6
White British	131	67.5	Student	14	7.2
White Other	22	11.3	Prefer not to say	4	2.1
Any Asian	10	5.2	Household income:		
Any Black	16	8.2	Under £5,199	2	1.0
Other	11	5.7	£5,200 - £10,399	4	2.1
Prefer not to say	4	2.1	£10,400 - £15,599	9	4.6
Country:			£15,600 - £ 19,799	11	5.7
England	173	89.2	£20,800 - £25,999	14	7.2
Scotland	11	5.7	£26,000 - £31,199	19	9.8
Wales	10	5.1.5	£31,200 - £36,399	18	9.3
			£36,400 - £51,999	29	14.9
			£52,000 and above	35	18.1
			Prefer not to say	53	27.3

Appendix S1. Focus group protocol

- Begin: *What was your general impression of this woodland*?
- Ask participants to discuss the attributes they noticed
    - Ensure all participants take turn (do not have to list everything)
    - Ask for reasoning behind each answer
    - Try to focus on natural elements. If non-natural elements are mentioned (e.g. fences, people, cars), that is okay as long as both natural and non-natural elements are covered in discussion
- If conversation takes off on various aspects, leave to develop
    - Probe on particular aspects of biodiversity participants notice
    - Probe on reactions to these aspects (e.g. valence)
    - If they give general answer, try to ask for more details. Use questions below as possible prompts if necessary:
        - *What stood out for you?*
        - *What do you think was missing from the woodland?*
        - *What did you find that you weren't expecting?*
        - *What elements do you personally look for in this kind of woodland?*
        - *Are some elements more preferable in the woodland?*
        - *Are some elements less preferable in the woodland?*
        - *Are some woodland elements associated with more positive emotions? Which?*
        - *Are some woodland elements associated with more negative emotions? Which?*
        - *Was there something others noticed that you wish you had? Why?*
        - *Was there something you noticed but wish you had not? Why?*
        - *Is there anything else you would like to mention?*
- Take care to reduce intimidation and aggression in groups
- Close the discussion and thank participants
- Turn off audio recording

Table S2. List of specific smells (*N*=284) mentioned by participants and their collective smell codes (*N*=103).

Smell code	Participant-mentioned smells
absence of smell	*absense of smells, couldn't smell anything, couldn't smell much, devoid of smell, didn't smell anything, hard to smell anything, lack of smell, lack of smells, less smells, less strong, no distinctive smell, no smell, no smells, not really smell anything, no prominent smells, no odour, no specific smell, no specific smells, not as many smells, not that many smells, nothing, nothing at all, nothing identifiable, nothing overpowering, nothing stood out, smell nothing, struggled with smelling, struggling to find some smells*
acidic	*acidic*
agricultural	*agricultural smell*
alive	*alive*
allergens	*allergens*
apple	*apple, sweet apple*
autumnal	*autumn smells, autumn-y, autumnal smell, autumn-ness smell*
bitter	*bitter*
bonfire	*bonfire*
bracken	*bracken'y*
burnt toffee	*burnt toffee*
cabbage	*cabbage*
Christmas	*Christmas, Christmassy*
clammy	*clammy*
citrus	*citrusy*
clean	*clean, clean air, cleaner, cleanliness, pure, pure air, untouched smell*
cold	*cold*
compost	*compost, composting, compost'y*
crisp	*crisp, crisper, crispness*
curry	*curry-ish*
damp	*damp, damp earth, damp leaf, damp leaves, damp smell, damp wood, dampness, vague dampness*
dark	*dark*
decay	*breaking down, dead leaves, decay, decaying, decomposed, decomposing, decomposing wood, mulching*
dew	*dew*
dirty	*dirty*
dry	*dryer*
dry wood	*dry wood*
dusty	*dusty*
earthy	*earth, earthiness, earthly, earthy, earthy smell*
elderflower	*elderflower*
eucalyptus	*eucalyptus*
faeces	*dog poo*
fermentation	*fermented, fermented leaves*
festering	*festering*

fire	*burning*
floral	*floral, flowers*
foliage	*foliage, forest, leafy, like a plant, undergrowth leaves, undergrowth'y, vegetation*
fox	*fox smell*
fox urine	*fox urine*
fragrant	*fragrance, fragrant*
fresh	*fresh, fresher, freshness, fresh smell, refreshing*
fresh air	*fresh air, fresh breeze*
fruity	*fruity*
fungi	*fungi, fungus, fungus'y, mushroom'y*
funky	*funky*
fusty	*fusty, fusty smell*
garden	*garden*
garlic	*garlic, garlic'y, hint of garlic, wild garlic*
gentle	*gentle*
grass	*fresh grass, grass smell, grassy*
green	*green*
herbal	*herbal smells, slight herbal smell*
hint of vinegar	*hint of vinegar*
homely	*homely smell*
humidity	*humidity*
humus	*humus*
leachate	*leachate*
leaf litter	*leaf litter*
lemon	*lemony*
light	*light*
mild	*mild*
mint	*mint, minty*
mould	*leaf mould, mouldiness, mouldy, mouldy wood*
moss	*moss*
mud	*fresh mud, mud, muddier, muddy, old wet mud, smell of mud*
muggy	*muggy*
musty	*musty, mustiness*
nature	*lack of urban smell, less polluted, natural, natural scents, natural smells, nature sort of smell, proper forest smell*
oaky	*oaky*
organic	*organic*
outdoors	*healthy outdoor smell, outdoor smell, outdoors*
ozone	*ozone*
peat	*peaty smell*
pefume	*perfume, perfumes*
petrichor	*petrichor*
pine	*fresh pine, pine, pine cones, pine needles, pines, pine'y*
rain	*rain, rain on fresh soil, raining, rainwater, real fresh wet rain smell, the smell when it rains on earth, thunderstorm*

rancid	*rancid*
raspberry	*raspberry*
rot	*rot, rotted, rotten, rotten wood, rotting, rotting leaf, rotting leaves*
rusty	*rusty*
sharp	*sharp, sharpness*
smell	*aroma, bit of a smell, change in the air, distinct smell, distinctive, distinctive aroma, distinctive smell, one smell, powerful, scent, scented, smell, smelly, smelt, smelt nice, something, whiff*
smells	*different things, smells, mixture of smells*
smoke	*smoke, smoky*
soil	*rich soil, soil*
spicy	*spicy*
stale	*stale*
sterile	*sterile*
spring	*spring*
strong smell	*prominent smell, pungent, significant smell, smell stronger, smelled quite strong, strong, strong smell*
summer	*summer smells*
sweet	*sweet, sweeter, sweetness*
tangy	*tangy*
tea	*tea*
tree bark	*bark*
trees	*smell of different trees, trees*
warmth	*warmth*
weak smell	*couldn't smell much, not much smell, slight, slight smell, slight scent, subtle*
wet	*fresh woodland wetness, moist, moisture, wet, wetness, water*
winter	*winter, winter smells*
wood	*wood, wood smell, woody*
woodland	*smelt like woodland, woodland, woodland smell, woods, woodsiness, woodsy*
